# Involvement of the Spinal Serotonergic System in the Analgesic Effect of [6]-Shogaol in Oxaliplatin-Induced Neuropathic Pain in Mice

**DOI:** 10.3390/ph16101465

**Published:** 2023-10-15

**Authors:** Juan Gang, Keun-Tae Park, Suyong Kim, Woojin Kim

**Affiliations:** 1 Department of East-West Medicine, Graduate School, Kyung Hee University, Seoul 02447, Republic of Korea; khan2296@naver.com; 2 Department of Physiology, College of Korean Medicine, Kyung Hee University, Seoul 02453, Republic of Korea; cerex@naver.com (K.-T.P.); tydsla123@naver.com (S.K.); 3 Korean Medicine-Based Drug Repositioning Cancer Research Center, College of Korean Medicine, Kyung Hee University, Seoul 02447, Republic of Korea

**Keywords:** [6]-shogaol, oxaliplatin, serotonin, spinal cord

## Abstract

Oxaliplatin is a chemotherapy drug that can induce severe acute neuropathy in patients within hours of treatment. In our previous study, 10 mg/kg [6]-shogaol (i.p.) significantly alleviated cold and mechanical allodynia induced by a 6 mg/kg oxaliplatin injection (i.p.); however, the precise serotonin-modulatory effect has not been investigated. In this study, we showed that intrathecal injections of NAN-190 (5-HT_1A_ receptor antagonist, 1 µg) and MDL-72222 (5-HT_3_ receptor antagonist, 15 µg), but not ketanserin (5-HT_2A_ receptor antagonist, 1 µg), significantly blocked the analgesic effect of [6]-shogaol (10 mg/kg, i.p.). Furthermore, the gene expression of the serotonin-synthesizing enzyme tryptophan hydroxylase 2 (TPH2) and serotonin levels in the spinal cord and serum were significantly downregulated (*p* < 0.0001 and *p* = 0.0002) and upregulated (*p* = 0.0298 and *p* = 0.0099) after oxaliplatin and [6]-shogaol administration, respectively. Moreover, both the gene and protein expression of the spinal serotonin receptors 5-HT_1A_ and 5-HT_3_ significantly increased after [6]-shogaol injections (*p* < 0.0001). Finally, intrathecal injections of both receptor agonists (8-OH-DPAT; 5-HT_1A_ receptor agonist, 10 µg and m-CPBG; 5-HT_3_ receptor agonist, 15 µg) mimicked the effects of [6]-shogaol in oxaliplatin-injected mice. Taken together, these results demonstrate that [6]-shogaol attenuates oxaliplatin-induced neuropathic pain by modulating the spinal serotoninergic system.

## 1. Introduction

Oxaliplatin is a third-generation platinum-based chemotherapeutic drug that is widely used to treat colorectal and breast cancers [1]. However, it can induce severe acute neuropathic pain in up to 65–98% of injected patients [2]. Although gabapentin and duloxetine [3] are widely used to alleviate pain, they have been reported to induce side effects such as nausea, dry mouth, headache, and dizziness [4,5]. Thus, to date, no optimal treatment has been presented, and efforts are underway to determine an optimal treatment. 

Previously, our laboratory focused on finding an optimal treatment to alleviate oxaliplatin-induced neuropathic pain [6,7,8,9]. As a potential candidate, *Zingiber officinale* Roscoe (*Z. officinale*) extract was suggested, as it could significantly alleviate cold and mechanical allodynia induced by oxaliplatin injection [7]. Furthermore, serotonergic receptors were shown to be involved, as the gene expression of the spinal 5-HT_1A_ receptor was significantly upregulated following *Z. officinale* injections. The major components of *Z. officinale* are known to be gingerol and shogaol. Thus, in our subsequent study, the effect of [6]-shogaol was assessed. We found that 10 mg/kg, but not 1 mg/kg, of [6]-shogaol could alleviate the cold and mechanical allodynia induced by oxaliplatin injections [6]. In that study, the role of [6]-shogaol in modulating the spinal serotonergic system and gamma-aminobutyric acid (GABA) was demonstrated, but changes in serotonin levels and in the gene and protein expression of serotonergic receptors in the spinal cord were not investigated. To date, only two studies have assessed the role of [6]-shogaol in pain [10,11]. In these studies, intraperitoneally administered [6]-shogaol decreased streptozotocin-induced heat and mechanical pain in mice. The doses used ranged from 5 to 15 mg/kg, and the effect of 10 mg/kg of [6]-shogaol was similar to that of 100 mg/kg of gabapentin. As the mechanism of action, sciatic nerve damage [10] and transient receptor potential vanilloid 1 (TRPV1) [11] have been assessed. However, although serotonin is known to be an important neurotransmitter in pain modulation [12] and serotonin-modulatory effect of *Z. officinale* and its components have been reported [13,14], whether [6]-shogaol can directly influence the serotonin level in the central nervous system (CNS) has never been investigated.

Serotonin (5-hydroxytryptamine, 5-HT) is a bioamine derived from the amino acid tryptophan. In serotonin-synthesizing cells, tryptophan is hydroxylated by tryptophan hydroxylase (TPH) and subsequently decarboxylated by aromatic acid decarboxylase (AADC) [15]. Serotonin is widely distributed in the central and peripheral nervous systems; however, the blood–brain barrier (BBB) is known to be impermeable to serotonin, and its role in the brain and periphery is known to be different [16]. Monoamines are not exchanged between the peripheral nervous system (PNS) and CNS, and serotonin in the PNS and CNS is encoded by two different genes (TPH1 and TPH2, respectively). Although 95% of the serotonin in the body is produced in the periphery and only 5% is produced in the brain, serotonin produced in the brain plays an important role in the regulation of mood, cognition, reward, learning, and pain [17,18,19]. During pain, it is mainly involved in descending pain inhibition [20] and attenuating ascending pain signals to the brain. In line with this, enhancing 5-HT [21] or modulating the function of its receptor in the spinal cord has been reported to decrease pain in various animal models of pain [19,22,23]. To date, seven families of serotonin receptors comprising 15 subtypes have been reported. Among them, 5-HT_1A_ and 5-HT_3_ receptors have been implicated in pain [24]. 

Some studies have reported that *Z. officinale* and its sub-components can modulate the serotonergic system [13,14], and in the PNS, *Z. officinale* was shown to mostly act as a serotonin inhibitor and 5-HT_3_ receptor antagonist [25,26]. However, in our previous study [6], serotonin receptor antagonists blocked the effect of [6]-shogaol, demonstrating that [6]-shogaol can also act as a serotonin receptor agonist in the CNS. Thus, the serotonin-modulatory effect of [6]-shogaol needs further clarification. 

In this study, we aimed to (1) clarify changes in serotonin levels in the spinal cord and serum, (2) measure changes in the serotonin-synthesizing enzyme TPH2 in the spinal cord, and (3) analyze the gene and protein expression of spinal 5-HT_1A_ and 5-HT_3_ receptors in mice with [6]-shogaol-treated oxaliplatin-induced neuropathic pain.

## 2. Results

### 2.1. [6]-Shogaol Alleviates Oxaliplatin-Induced Cold and Mechanical Allodynia in Mice

**Figure 1:**
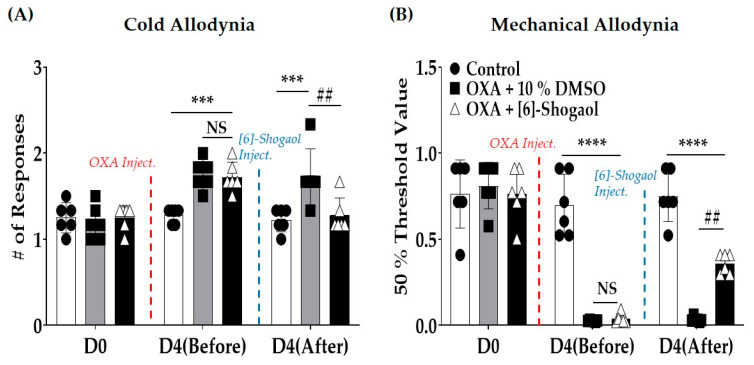
Intraperitoneal injection of [6]-shogaol attenuated oxaliplatin-induced neuropathic pain in mice. Single oxaliplatin injection (i.p.) induced cold (**A**) and mechanical (**B**) allodynia. Cold and mechanical allodynia were assessed using acetone drop (**A**) and von Frey filament (**B**) tests, respectively. Behavioral tests were conducted before the injection of oxaliplatin (D0), four days after oxaliplatin injection (but before the injection of 10% dimethyl sulfoxide (DMSO) or [6]-shogaol (D4 (Before)), and on the same day, one hour after intraperitoneal injection of 10 mg/kg [6]-shogaol or 10% DMSO (D4 (After)). Data are presented as mean ± standard deviation (SD). Control: *n* = 6; OXA + 10% DMSO: *n* = 6; OXA + [6]-Shogaol: *n* = 6. D: day; DMSO: dimethyl sulfoxide; Inject.: injection; NS: non-significant; OXA: oxaliplatin. *** *p* < 0.001, **** *p* < 0.0001: Control vs. OXA + 10% DMSO or OXA + [6]-Shogaol; ## *p* < 0.01: OXA + 10% DMSO vs. OXA + [6]-Shogaol with two-way ANOVA followed by Tukey’s multiple comparisons test.

A single oxaliplatin injection induced acute cold (Figure 1A) and mechanical allodynia (Figure 1B) in the mice (D4 (Before)—Control vs. OXA + 10% DMSO: Cold Allodynia: *p* = 0.0002; Mechanical Allodynia: *p* < 0.0001). However, a single intraperitoneal administration of 10 mg/kg [6]-shogaol significantly alleviated allodynia when measured one hour after injection (D4 (After)—OXA + 10% DMSO vs. OXA + [6]-Shogaol: Cold Allodynia: *p* = 0.0005; Mechanical Allodynia: *p* = 0.0001). The dose of [6]-shogaol was set based on our previous study, in which 10 mg/kg was more effective than 1 mg/kg against oxaliplatin-induced neuropathic pain [6]. The effect of [6]-shogaol was stronger against cold than mechanical allodynia, as the number of responses to acetone drop decreased to the level of the control. In mechanical allodynia, [6]-shogaol failed to increase the threshold to that of the control level. Cold and mechanical allodynia were assessed using the acetone drop and von Frey filament tests, respectively.

### 2.2. Intrathecal Injections of Serotonin Receptor Antagonists Prevent the Analgesic Effect Induced by [6]-Shogaol Injections

**Figure 2:**
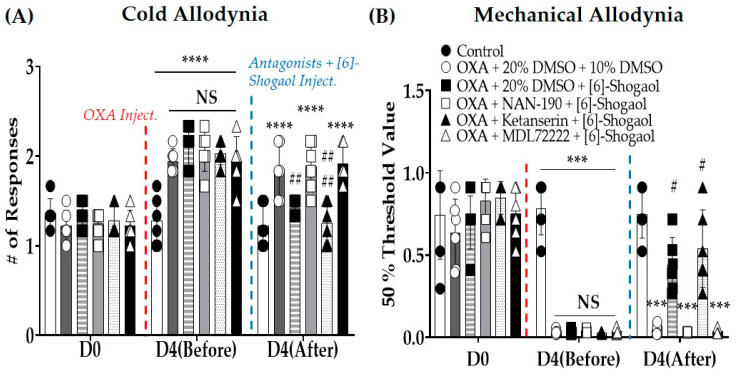
Effect of intrathecal injection of serotonin receptor _1A_, _2A,_ and _3_ antagonists on the analgesic effect of [6]-shogaol against oxaliplatin-induced cold (**A**) and mechanical (**B**) allodynia. NAN-190, ketanserin, and MDL-72222 are serotonin _1A_, _2A,_ and _3_ receptor antagonists, respectively. Cold allodynia was assessed using the acetone drop test (**A**), and mechanical allodynia was measured using the von Frey filament test (**B**). All groups, except the control, received oxaliplatin injection four days prior to the behavior test. As a control for NAN-190, ketanserin, and MDL-7222220%, dimethyl sulfoxide (DMSO) was used, and 10% DMSO was used as a control for [6]-Shogaol. We administered 10% DMSO or [6]-shogaol intraperitoneally 20 min after intrathecal injection of 20% DMSO, NAN-190, ketanserin, or MDL-72222. D0: before injection of oxaliplatin. D4 (Before): before the injection of 10% and 20% DMSO, NAN-190, ketanserin, MDL-72222, and [6]-shogaol. D4 (After): 1 h after injection of 10% and 20% DMSO, NAN-190, ketanserin, MDL-72222, and [6]-shogaol. Data are presented as mean ± SD. Control: *n* = 6; OXA + 20% DMSO + 10% DMSO: *n* = 6; OXA + 20% DMSO + [6]-Shogaol: *n* = 6; OXA + NAN-190 + [6]-Shogaol: *n* = 6; OXA + Ketanserin + [6]-Shogaol: *n* = 6; OXA + MDL-72222 + [6]-Shogaol: *n* = 6. D: day; DMSO: dimethyl sulfoxide; Inject.: injection; NS: non-significant; OXA: oxaliplatin. *** *p* < 0.001, **** *p* < 0.0001 vs. Control; # *p* < 0.05, ## *p* < 0.01, #### *p* < 0.0001 vs. OXA + 20% DMSO + 10% DMSO with two-way ANOVA followed by Tukey’s multiple comparisons test.

To confirm the role of spinal serotonergic receptors in the analgesic effect of [6]-shogaol, 5-HT_1A_, _2A_, and _3_ receptor antagonists were administered intrathecally 20 min before the injection of 10 mg/kg [6]-shogaol in mice with oxaliplatin-induced neuropathic pain (Figure 2A,B). NAN-190 (5-HT_1A_ receptor antagonist, 1 μg/mouse, concentration 0.2 μg/μL), ketanserin (5-HT_2A_ receptor antagonist, 1 μg/mouse, concentration 0.2 μg/μL), and MDL-72222 (5-HT_3_ receptor antagonist, 15 μg/mouse, concentration 3 μg/μL) were used as antagonists. The results showed that 5-HT_1A_ and _3_ (D4 (After)—OXA + NAN-190 + [6]-Shogaol vs. OXA + 20% DMSO + 10% DMSO: Cold Allodynia: *p* = NS; Mechanical Allodynia: *p* = NS. OXA + MDL72222 + [6]-Shogaol vs. OXA +20% DMSO + 10% DMSO: Cold Allodynia: *p* = NS; Mechanical Allodynia: *p* = NS), but not 5-HT_2A_ receptor antagonists (OXA + Ketanserin + [6]-Shogaol vs. OXA +20% DMSO + 10% DMSO: Cold Allodynia: *p* < 0.0001; Mechanical Allodynia: *p* = 0.0240), completely blocked the analgesic effects of [6]-shogaol on both cold and mechanical allodynia.

### 2.3. Spinal Serotonin Level Increases after [6]-Shogaol Administration

**Figure 3:**
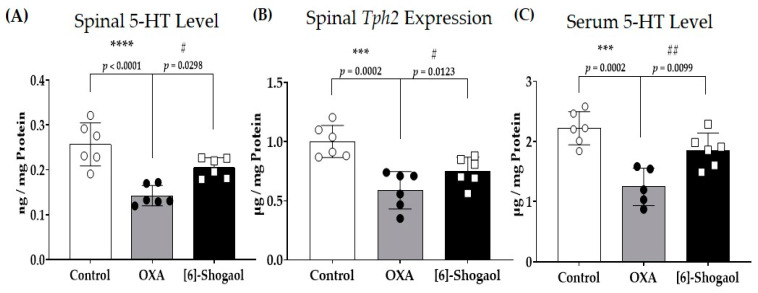
The effect of [6]-shogaol on spinal serotonin and relative tryptophan hydroxylase 2 (*Tph2*) gene expression level in oxaliplatin-injected mice. Changes in spinal serotonin level after oxaliplatin and [6]-shogaol injection (**A**). Changes in spinal *Tph2* gene expression after oxaliplatin and [6]-shogaol injection (**B**). Changes is serum serotonin level after oxaliplatin and [6]-shogaol injection (**C**). The level of 5-hydroxytryptamine (5-HT) was measured via enzyme-linked immunosorbent assay (ELISA). *Tph2* expression was measured using quantitative real-time polymerase chain reaction (RT-qPCR). Experiments were conducted on the fourth day prior to the injection of oxaliplatin when allodynia was induced in mice. The lumbar 4–5 spinal cords were sampled for the experiment. The control group received 5% glucose. The OXA group received 6 mg/kg of oxaliplatin and 10% of dimethyl sulfoxide (DMSO). The [6]-Shogaol group received oxaliplatin and 10 mg/kg [6]-shogaol. Data are presented as mean ± SD. Control: *n* = 6; OXA: *n* = 6; [6]-Shogaol: *n* = 6. *** *p* < 0.001, **** *p* < 0.0001: Control vs. OXA. # *p* < 0.05; ## *p* < 0.01: OXA vs. [6]-Shogaol with one-way ANOVA followed by Tukey’s multiple comparisons test.

*Z. officinale* is known to affect the serotonergic system in the body [7]. Serotonin levels in the spinal cord (Figure 3A) and serum (Figure 3C) and the gene encoding tryptophan hydroxylase 2 (TPH2) (Figure 3B) in the spinal cord were assessed. The TPH2 gene encodes the rate-limiting enzyme of serotonin synthesis in serotonergic neurons in the central nervous system (CNS) [27]. It is also the main factor that determines serotonin levels in the CNS [28]. The serotonin level, measured via enzyme-linked immunosorbent assay (ELISA), decreased after oxaliplatin injection and increased after [6]-shogaol administration both in the spinal cord and the serum. Furthermore, the gene expression of TPH2 also decreased and increased after oxaliplatin and [6]-shogaol injections, respectively. These results show that [6]-Shogaol can increase serotonin levels in the spinal cord by increasing its synthesis in the spinal cord. 

### 2.4. Spinal Serotonergic Receptors Are Involved in the Analgesic Effect of [6]-Shogaol

**Figure 4:**
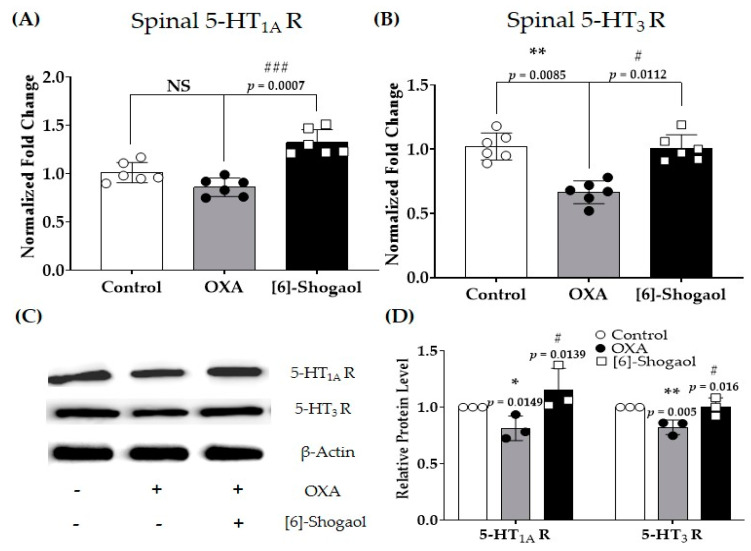
Changes in the serotonergic receptors in the spinal cord after oxaliplatin and [6]-Shogaol injections in mice. The gene (**A**,**B**) and protein (**C**,**D**) expressions of 5−hydroxytryptamine (5−HT)_1A_ and _3_ receptors were measured using reverse transcription–quantitative polymerase chain reaction (RT−qPCR) and Western blot, respectively. The lumbar 4–5 spinal cords were sampled for the experiment. The control group received 5% glucose. The OXA group received 6 mg/kg of oxaliplatin and 10% of dimethyl sulfoxide (DMSO). The [6]-Shogaol group received 6 mg/kg of oxaliplatin with 10 mg/kg of [6]-shogaol. All drugs were injected intraperitoneally. Data are presented as mean ± SD. Control: *n* = 6; OXA: *n* = 6; OXA + [6]-Shogaol: *n* = 6. 5−HT_1A_R: 5−HT_1A_ Receptor; 5−HT_3_R: 5−HT_3_ Receptor; NS: non-significant; OXA: oxaliplatin. * *p* < 0.05, ** *p* < 0.01: Control vs. OXA; # *p* < 0.05, ### *p* < 0.001: OXA vs. [6]-Shogaol with one-way ANOVA followed by Tukey’s multiple comparisons test.

As serotonin levels in the spinal cord changed after both oxaliplatin and [6]-shogaol injections, serotonergic receptors were assessed in the next experiment. The gene (Figure 4A,B) and protein (Figure 4C,D) expression levels of serotonergic receptors present in the spinal cord were assessed. The results showed that the gene expression of 5-HT_1A_ receptors (Figure 4A), but not that of 5-HT_3_ receptors (Figure 4B), was significantly downregulated after oxaliplatin injection. However, [6]-Shogaol (10 mg/kg, i.p.) significantly increased the gene expression of both 5-HT_1A_ and _3_ receptors in the spinal cord. To further confirm the changes in spinal cord receptors, Western blotting was performed (Figure 4C,D). The protein expression of both 5-HT receptors was downregulated compared to the control after oxaliplatin injection, whereas 10 mg/kg [6]-shogaol treatment significantly upregulated the expression of both receptors, as shown by the quantitative real-time polymerase chain reaction (RT-qPCR).

### 2.5. Intrathecal Injections of Serotonin Receptor Agonists Decrease Oxaliplatin-Induced Cold and Mechanical Allodynia in Mice

**Figure 5:**
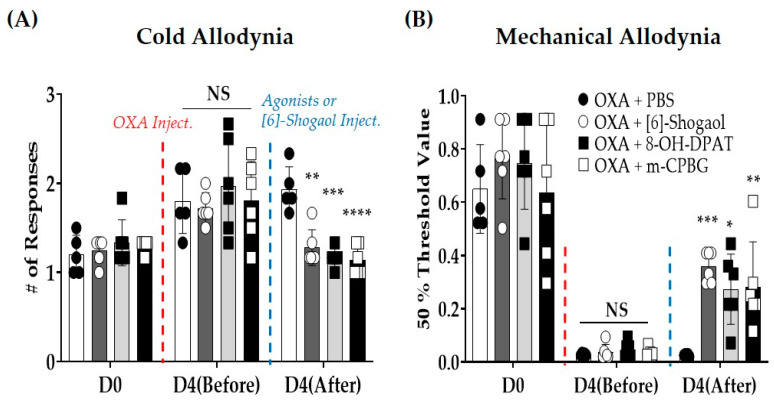
Effect of intrathecal administration of serotonin receptor _1A_ and _3_ agonists on oxaliplatin-induced cold (**A**) and mechanical (**B**) allodynia. (±)-8-hydroxy-2-(di-*n*-propylamino) tetralin hydrobromide (8-OH-DPAT) and 1-(m-chlorophenyl)-biguanide (m-CPBG) are serotonin _1A_ and _3_ receptor agonists, respectively. 8-OH-DPAT (10 μg/mouse, concentration 1 μg/μL) and m-CPBG (15 μg/mouse, concentration 1.5 μg/μL) were injected intrathecally. Cold allodynia was assessed using the acetone drop test (**A**) and mechanical allodynia was measured using the von Frey filament test (**B**). Phosphate-buffered saline (PBS) was used as a control for both 8-OH-DPAT and m-CPBG. D0: before injection of oxaliplatin. D4 (Before): four days after injection of oxaliplatin. D4 (After): 30 min after injection of 5-HT receptor agonists. Data are presented as mean ± SD. D: day; DMSO: dimethyl sulfoxide; Inject.: injection; NS: non-significant; OXA: oxaliplatin; PBS: phosphate-buffered saline; OXA + PBS: *n* = 5; OXA + [6]-Shogaol: *n* = 6; OXA + 8-OH-DPAT: *n* = 5; OXA + m-CPBG: *n* = 6. * *p* < 0.05, ** *p* < 0.01, *** *p* < 0.001, **** *p* < 0.0001 vs. OXA + PBS with two-way ANOVA followed by Tukey’s multiple comparisons test.

The results showed that spinal 5-HT_1A_ and _3_ receptors were involved in the analgesic effect of [6]-shogaol, and behavioral tests were conducted after the intrathecal administration of 5-HT receptor agonists (Figure 5A,B). The 5-HT_1A_ and _3_ receptor agonists are (±)-8-hydroxy-2-(di-*n*-propylamino) tetralin hydrobromide (8-OH DPAT, 10 μg/mouse, concentration 1 μg/μL, i.t.) and 1-(m-chlorophenyl)-biguanide (mCPBG, 15 μg/mouse, concentration 1.5 μg/μL, i.t.), respectively. One group was treated with [6]-shogaol (10 mg/kg, i.p.) for comparison. The results show that both 5-HT_1A_ and _3_ receptor agonists alleviated cold and mechanical allodynia induced by oxaliplatin injection in mice (D4 (After)—Cold Allodynia: OXA + PBS vs. OXA + [6]-Shogaol: *p* = 0.0013, vs. OXA + 8-OH-DPAT: *p* = 0.0001, vs. OXA + m-CPBG: *p* < 0.0001; Mechanical Allodynia: OXA + PBS vs. OXA + [6]-Shogaol: *p* = 0.0005, vs. OXA + 8-OH-DPAT *p* = 0.0133, vs. OXA+ m-CPBG: *p* = 0.0094). The behavioral responses to acetone drops and von Frey filaments were similar to those in [6]-shogaol-injected mice.

## 3. Discussion

In this study, the changes in spinal serotonin levels after [6]-shogaol administration were demonstrated for the first time. Serotonin and its synthesizing enzyme (i.e., TPH2) were downregulated and upregulated, respectively, after oxaliplatin and [6]-shogaol injections. Furthermore, the gene and protein expression of serotonergic receptors were altered after [6]-shogaol injection, and intrathecal injections of these receptor agonists and antagonists confirmed their role in oxaliplatin-induced neuropathic pain.

### 3.1. [6]-Shogaol and Spinal Serotonin Synthesis

In our previous study, we demonstrated the role of serotonin receptors in GABA activation; however, quantification of the spinal serotonergic system (i.e., serotonin and serotonergic receptors) had not been conducted. In various types of pain, the involvement of the serotonergic system has been reported [12,19,29], and increased activity of spinal cord serotonin neurons was reported to be associated with an analgesic effect [30]. However, the serotonin-modulating effect of [6]-shogaol is not well understood in pain. In this study, spinal serotonin and its rate-limiting synthesizing enzyme, TPH2, significantly increased after the injection of 10 mg/kg [6]-shogaol (*p* = 0.0123). In the central nervous system (CNS), serotonin acts as a neurotransmitter and is mainly produced in the raphe nuclei of the brain stem [31]. Although most of the serotonergic neurons are located in the brainstem, a study conducted with a T4–T5 transacted rat showed that the spinal cord also has some serotonin-producing neurons, as 2–10% of 5-HT-like immunoreactive neurons remained after transection [32]. Thus, because [6]-shogaol is known to easily penetrate the BBB via passive diffusion [33], the increase in the gene expression of TPH2 shows that [6]-shogaol increases the synthesis of serotonin in the spinal cord. TPH2 and TPH1 are both enzymes that are needed in 5-HT synthesis; however, TPH2 is mainly expressed in the CNS, whereas TPH1 is in the peripheral nervous system (PNS) [27].

### 3.2. Serotonergic Receptors and [6]-Shogaol-Induced Analgesia

In this study, the spinal serotonergic receptors 5-HT_1A_ and _3_, but not 5-HT_2A_, were shown to be involved in the effects of [6]-Shogaol. 5-HT_2_ receptors are reported to be less numerous in the spinal cord than other serotonergic receptors, such as 5-HT_1A_ or _3_ [34]. Furthermore, even in the spinal cord, they are located more in the ventral than in the dorsal part [35], suggesting their low involvement in sensory signaling. As mentioned in the Introduction, 5-HT_1A_ and _3_ receptors have different properties, as 5-HT_1A_ receptors are GPCRs, whereas 5-HT_3_ receptors are ligand-gated ion channels. Moreover, the 5-HT_1A_ receptor is coupled with Gi/o, suggesting an inhibitory effect [36], whereas the 5-HT_3_ receptor is directly linked to nonselective cationic channels, suggesting an excitatory effect [37]. G_i/o_-coupled GPCRs are known to stimulate G-protein-activated inwardly rectifying K^+^ channels (GIRK) through direct interactions between G_βγ_ and the channel, leading to hyperpolarization and thus decreased neuronal excitability [38]. In an electrophysiological study conducted by Abe et al. [36], 5-HT application induced an outward current in excitatory substantia gelatinosa neurons in the rat spinal cord, suggesting an inhibitory effect of serotonin in the spinal cord. This outward current induced by 5-HT could be mimicked by 5-HT_1A_, but not _2_ and _3_ receptor agonists (i.e., (±)-8-hydroxy-2-(di-*n*-propylamino) tetralin hydrobromide (8-OH-DPAT), (±)-1-(2,5-dimethoxy4-iodophenyl)-2-aminopropane hydrochloride (DOI), and 1-(m-chlorophenyl)-biguanide (m-CPBG)). However, the 5-HT_3_ receptor agonist (m-CPBG) induced an increase in the frequency and amplitude of spontaneous inhibitory postsynaptic currents (sIPSCs) [36]. Altogether, these results suggest an inhibitory effect of 5-HT_1A_ receptors on excitatory neurons and an excitatory effect of 5-HT_3_ receptors on inhibitory neurons in the spinal cord. 

In our study, in accordance with the above results, the intrathecal injection of both 5-HT_1A_ and 5-HT_3_ receptor agonists (i.e., 8-OH DPAT and mCPBG) significantly alleviated pain (Figure 5: against cold allodynia, *p* = 0.0001 and *p* < 0.0001, respectively; against mechanical allodynia, *p* = 0.0133 and *p* = 0.0094, respectively), and both receptor antagonists blocked the effect of [6]-shogaol on oxaliplatin-induced neuropathic pain (Figure 2: against cold and mechanical allodynia, both NAN-190 and MDL-72222 pre-treated group of mice showed no difference compare to oxaliplatin-treated group of mice). Although in our study, the effect of [6]-shogaol on excitatory or inhibitory neurons was not assessed, [6]-shogaol significantly increased spinal GABA (*p* = 0.04) (Supplementary Figure S1); in our previous study, it increased the number of GABA-positive neurons in the spinal dorsal horn of oxaliplatin-treated mice. However, further well-designed studies should be conducted to clearly understand the role of [6]-shogaol in spinal 5-HT_1A_ and 5-HT_3_ receptors.

### 3.3. Role of [6]-Shogaol and Future Studies

[6]-Shogaol is the dehydrated form of gingerol and has been reported to have many biological effects [39]. Both in vivo and in vitro studies have reported that [6]-shogaol has anti-inflammatory, anti-bacterial, and anti-oxidative properties [40], as reduced white blood cell infiltration into the inflamed tissue and decreased inflammatory mediator such as cyclooxygenase-2 (COX-2) were reported [41]. In addition, [6]-shogaol is also known to have an anti-obesity effect. The molecular target and pathways of the anti-obesity effect of [6]-shogaol have been verified by using network pharmacology [42], and 86 core targets of [6]-shogaol have been identified. Many studies have also reported the effect of [6]-shogaol in nausea and vomiting [43]. It was reported to mimic the activity of 5-HT antagonists (e.g., ondansetron) in the peripheral nervous system (PNS) [13,25]. Thus, the results presented in this study imply that [6]-shogaol may have two different serotonin-modulatory effects in the PNS and CNS. In the CNS, [6]-shogaol may act as a serotonin receptor agonist and increase the serotonin level, whereas in the PNS, [6]-shogaol can act as serotonin receptor antagonist [25]. However, with regard to its role in pain, the number of papers is too small [44]. Thus, sophisticated pharmacodynamics, preclinical pharmacokinetics, bioavailability, and toxicity studies are needed to clarify the role and action of [6]-shogaol in pain.

## 4. Materials and Methods

### 4.1. Animals

Adult C57BL/6 mice (7 weeks old) were obtained from Daehan Biolink (Chungbuk, Republic of Korea). They were housed in a specific pathogen-free animal center where the temperature and humidity were maintained at 23 ± 2 °C and 65 ± 5%, respectively. A 12 h light/dark cycle was maintained, and food and water were provided ad libitum. All experimental protocols were approved by the Kyung Hee University Animal Care and Use Committee (KHUASP(SE)-21-543) on 2 February, and were conducted in accordance with the guidelines of the International Association for the Study of Pain.

### 4.2. Behavioral Tests

To assess behavioral changes in the mice, acetone drop and von Frey filament tests were conducted to measure cold and mechanical allodynia, respectively, as in our previous studies [45,46]. All animals were placed on a metal mesh floor and caged in an inverted clear plastic cage (12 × 8 × 6 cm^3^) for 30 min before all measurements were taken for acclimation. Mice were randomly allocated into three groups to assess the effect of oxaliplatin and [6]-shogaol (Figure 1—Control: *n* = 6; OXA + 10% DMSO: *n* = 6; OXA + [6]-Shogaol: *n* = 6). 

#### 4.2.1. Measurement of Cold Allodynia

To measure responses to cold stimuli, an acetone drop (10 μL) was applied on each mid-plantar hind paw of the mice. Acetone drops were applied three times to both paws, and licking and shaking in response to the acetone drops were observed for 30 s [47]. The “# of responses” in the *Y*-axis refers to the average number of responses to six instances of the assay.

#### 4.2.2. Measurement of Mechanical Allodynia

To measure responses to mechanical stimuli, a series of von Frey filaments (bending forces of 0.02, 0.04, 0.07, 0.16, 0.4, 0.6, 1, 1.4, and 2 g, Stoelting, Kiel, WI, USA) were applied on the mid-plantar hind paws and the the up-and-down method was used to measure the responses [48]. First, a filament with a bending force of 0.16 g was applied perpendicular to the plantar surface of the hind paw and pressed until bent for two to three seconds. Whenever a response (withdrawal, licking, or biting of the paw) to stimulation occurred, a filament with a lower bending force (i.e., 0.07 g) was applied. However, if no response as observed, a filament with a higher bending force (i.e., 0.4 g) was applied to the paw. Five stimuli were applied for each bending force, resulting in a certain pattern of positive and negative responses [48]. Using Dixon’s up–down method [49] and Chaplan’s calculation method [50], average values were obtained for both hind paws. The 50% threshold value on the *y*-axis refers to the average number of responses for both hind paws. The advantages of the up-and-down method are that only a small sample size is required to estimate the efficacy and it has a simple study design. However, some limitations are also present as only responses to acute stimuli and not spontaneous pain can be assessed.

### 4.3. Oxaliplatin Injection

**Figure 6:**
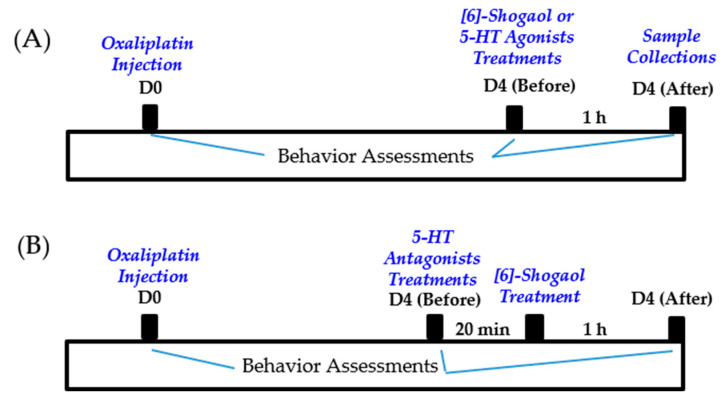
Timeline of behavior assessments conducted for [6]-shogaol and 5-HT agonists treatments shown in Figure 1 and Figure 5 (**A**). Timeline of the effect of 5-HT antagonists treatments in [6]-shogaol induced analgesic effect on oxaliplatin-induced neuropathic pain, demonstrated in Figure 2 (**B**).

Oxaliplatin (Sigma-Aldrich, St. Louis, MO, USA) was dissolved in a 5% glucose solution at a concentration of 2 mg/mL, as in our previous study [6]. The dissolved oxaliplatin was administered intraperitoneally at a dose of 6 mg/kg. The mice in the control group were injected with the same amount of 5% glucose. As in our previous experiments, cold and mechanical allodynia were significantly induced two to six days after injection. All experiments were conducted on day four when cold and mechanical allodynia were strongly induced [7] (Figure 6).

### 4.4. Administration of [6]-Shogaol

[6]-Shogaol (FUJIFILM Wako Pure Chemical Corporation, Osaka, Japan) was dissolved in a 10% dimethyl sulfoxide (DMSO) solution at a concentration of 1.5 mg/mL as in our previous study [6]. [6]-Shogaol was injected intraperitoneally at a dose of 10 mg/kg. As a control, the same volume (0.2 mL) of 10% DMSO was injected. [6]-Shogaol or 10% DMSO was administered four days (D4) after oxaliplatin injection (Figure 6A).

### 4.5. Intrathecal Injection

#### 4.5.1. 5-HT Antagonist Administration

To clearly assess the role of spinal 5-HT receptors in the analgesic effect of [6]-shogaol, NAN-190 (5-HT_1A_ receptor antagonist, 1 μg/mouse, concentration 0.2 μg/μL), ketanserin (5-HT_2A_ receptor antagonist, 1 μg/mouse, concentration 0.2 μg/μL), and MDL-72222 (5-HT_3_ receptor antagonist, 15 μg/mouse, concentration 3 μg/μL) were intrathecally injected 20 min before the administration of [6]-shogaol (Figure 6B). NAN-190, ketanserin, and MDL-72222 were dissolved in 20% DMSO, respectively. Thus, the control group received 20% DMSO (i.t.). Behavioral assessments were conducted twice (before the injection of 5-HT receptor antagonists and 1 h after [6]-shogaol treatment) (Control: *n* = 6; OXA + 20% DMSO + 10% DMSO: *n* = 6; OXA + 20% DMSO + [6]-Shogaol: *n* = 6; OXA + NAN-190 + [6]-Shogaol: *n* = 6; OXA + Ketanserin + [6]-Shogaol: *n* = 6; OXA + MDL-72222 + [6]-Shogaol: *n* = 6).

#### 4.5.2. 5-HT Agonist Administration

In addition, to confirm the effect of spinal 5-HT receptor activation, 8-OH-DPAT (5-HT_1A_ receptor agonist, 10 μg/mouse, concentration 1 μg/μL), and m-CPBG (5-HT_3_ receptor agonist, 15 μg/mouse, concentration 1.5 μg/μL) were injected intrathecally (Figure 6A). 8-OH-DPAT and m-CPBG were dissolved in PBS, whereas the control group received PBS. 5-HT receptor antagonists and agonists were purchased from Tocris (Cookson, UK). Intrathecal injection of antagonist solutions (5 μL) and agonist solutions (10 μL) were performed at the lumbar 4–5 intervertebral level using a Hamilton syringe (Hamilton Company, Reno, NV, USA) after isoflurane anesthesia (OXA + PBS: *n* = 5; OXA + [6]-Shogaol: *n* = 6; OXA + 8-OH-DPAT: *n* = 5; OXA + m-CPBG: *n* = 6).

### 4.6. Tissue Preparation

Four days after oxaliplatin administration when pain was induced, the mice were anesthetized with isoflurane and transcardially perfused with 0.1 M phosphate-buffered saline (PBS) (Figure 6A). After perfusion, lumbar 4–5 spinal cord segments were collected and were frozen and stored at −80 °C for quantitative real-time polymerase chain reaction (qRT-PCR), enzyme-linked immunosorbent assay (ELISA), and Western blot. The data are presented in Figure 3 and Figure 4, and six mice were used per group (Control: *n* = 6; OXA: *n* = 6; OXA + [6]-Shogaol: *n* = 6).

### 4.7. Reverse Transcription–Quantitative Polymerase Chain Reaction (RT-qPCR)

Total ribonucleic acid (RNA) was extracted using an AccuPrep Universal RNA Extraction Kit (Bioneer, Daejeon, Republic of Korea) according to the manufacturer’s protocol. RNA concentration was quantified using a NanoDrop ND-1000 Spectrophotometer (Thermo Fisher Scientific, Waltham, MA, USA). cDNA was prepared using Maxime RT Premix (Intronbi, Seongnam, Korea). qPCR was performed using a SensiFAST SYBR No-ROX kit (Bioline, London, UK) and a CFX Connect Real-time PCR Detection System (Bio-Rad, CA, USA). The oligonucleotide primers used for the PCR were as follows: GAPDH forward 5′-GGA GGT AGC TCC TGA TTC GC-3′ and reverse 5′-CAC ATT GGG GGT AGG AAC AC-3′; 5-hydroxytryptamine receptor 1A (*Htr1a*) forward 5′-AAC TAT CTC ATC GGC TCC TT-3′ and reverse 5′-GAT TGC CCA GTA CCT GTC TA-3′; 5-hydroxytryptamine receptor 3A (*Htr3a*) forward 5′-TGG TCC TAG ACA GAA TAG GG-3′ and reverse 5′-GGT CTT CTC CAA GTC CTG A-3′; and tryptophan hydroxylase 2 (*Tph2*) forward 5′-CAT TCC TCG CAC AAT CCA GTC G-3′ and reverse 5′-CTT GAC ATA TTC AAC TAG ACG CTC-3. The reaction was preheated for 10 min at 94 °C followed by 40 cycles at 94 °C for 20 s, 57 °C for 20 s, and 72 °C for 30 s. GAPDH primers were used to standardize the amount of RNA in each sample. A reaction without cDNA was used as the negative control. The qPCR data were required for amplification-generated fluorescence to reach a specific threshold of detection (Ct value). Relative gene expression was quantified on the basis of an equal amount of RNA (0.1 μg) and the average Ct value for each gene [51]. Delta Ct (_Δ_Ct = Ct _target gene_ − Ct _reference gene_) was calculated using Ct values for the gene in the same sample. Actin was used as an internal reference control. The _ΔΔ_ Ct value was obtained via calculation using the equation _ΔΔ_Ct = (_Δ_Ct _target gene_ − _Δ_Ct _untreated_). The normalized expression fold was expressed as the value of 2^−ΔΔCt^ (GAPDH control = 1).

### 4.8. Enzyme-Linked Immunosorbent Assay (ELISA)

The animals were anesthetized via isoflurane inhalation, and spinal cord tissues were collected. To minimize tissue degradation, intracardial perfusion was performed using 20 mL cold PBS (pH 7.2). The prepared tissues were homogenized with radioimmunoprecipitation (RIPA) buffer (Thermo Fisher Scientific, Waltham, MA, USA) and a phosphatase inhibitor cocktail (100×, Thermo Fisher Scientific) [52]. The homogenized samples were incubated on ice for 15 min and centrifuged at 13,000 rpm at 4 °C for 20 min. The supernatant was collected in a fresh Eppendorf tube. To measure serotonin and GABA levels in the spinal cord, serotonin and GABA ELISA kits (LDN GmbH, Nordhorn, Germany) were used according to the manufacturer’s instructions (LDN GmbH, Nordhorn, Germany and MyBioSource, San Diego, CA, USA).

### 4.9. Western Blots

Western blotting was performed as previously described with minor modifications [53,54]. Spinal cord tissues were removed, placed in RIPA buffer, and homogenized using a bead beater. The proteins were quantified using a UV spectrophotometer. Each protein sample was adjusted to a protein concentration of 20 μg and subjected to 10 5 SDS-PAGE electrophoresis. After electrophoresis, the proteins were transferred to polyvinylidene fluoride membranes. The membrane was blocked with 5% non-fat milk in TBS-T/Tween 20 (TBS-T) for 1 h. The membrane was washed with TBS-T and left to be shaken overnight at 4 °C with primary antibody (β-actin, 1:2000, Invitrogen/5-HT_1A_, 1:1000, Novus Biologicals/5-HT_3A_, 1:1000, Novus Biologicals, Littleton, CO, USA) in 5% skim milk in TBS-T. The secondary antibody was incubated for 1 h at room temperature with goat anti-rabbit IgG HRP peroxidase (1:5000, Thermo Fisher Scientific, Waltham, MA, USA). Western blot bands were detected using an ECL solution (D-Plus ECL Femto, Hwaseong, Republic of Korea), and images were acquired using chemiluminescence. The bands were quantified using ImageJ software (version 1.8.0). Target bands were normalized to the amount of actin band.

### 4.10. Statistical Analysis

All data are presented as mean ± standard deviation (SD). Statistical analyses and graphic works were performed using Prism 7.0 (GraphPad Software, La Jolla, CA, USA). Two-way analysis of variance (ANOVA), followed by Tukey’s post hoc tests for multiple comparisons and Student’s *t*-tests, were used for statistical analyses. Statistical significance was set at *p* < 0.05.

## 5. Conclusions

In conclusion, these results show that 10 mg/kg [6]-shogaol alleviates oxaliplatin-induced neuropathic pain by increasing spinal serotonin levels. In addition, spinal 5-HT_1A_ and _3_, but not _2A_ receptors, were shown to be involved in the effect of [6]-shogaol, as pretreatment with their antagonist blocked the anti-allodynic effect. Furthermore, the gene and protein expression of these receptors were upregulated after [6]-shogaol treatment in the spinal cord, suggesting that [6]-shogaol modulates the spinal serotonergic system to attenuate oxaliplatin-induced neuropathic pain. To fully elucidate the role of the serotonin-modulatory effect of [6]-shogaol in oxaliplatin-induced neuropathic pain, future studies need to focus on the brain, especially the rostral ventromedial medulla (RVM), where most of the serotonin in the CNS is synthesized.

## Data Availability

Data is contained within the article and Supplementary Material.

## Supplementary Materials

The following supporting information can be downloaded at: https://www.mdpi.com/article/10.3390/ph16101465/s1, Figure S1: The effect of [6]-shogaol on spinal *gamma*-aminobutyric acid (GABA) expression level in oxaliplatin-injected mice. The level of GABA was measured by *enzyme-linked immunosorbent assay* (ELISA). Experiments were conducted on the fourth day prior to the injection of oxaliplatin when allodynia was induced in mice. The lumbar 4–5 spinal cords were sampled for experiment. The control group received 5% glucose. The OXA group received 6 mg/kg of oxaliplatin and 10% of dimethyl sulfoxide (DMSO). The [6]-Shogaol group received oxaliplatin and 10 mg/kg [6]-shogaol. Data are presented as mean ± SD. Control: *n* = 6; OXA: *n* = 6; [6]-Shogaol: *n* = 6. ** *p* < 0.01: Control vs. OXA. # *p* < 0.05: OXA vs. [6]-Shogaol with one-way ANOVA followed by Tukey’s multiple comparisons test.
