# Supplementary material for: Involvement of the Spinal Serotonergic System in the Analgesic Effect of [6]-Shogaol in Oxaliplatin-Induced Neuropathic Pain in Mice
Source: Pharmaceuticals (Basel). 2023 Oct 15;16(10):1465. doi: 10.3390/ph16101465 (PMC10610466; doi:10.3390/ph16101465)
Supplement: Supplementary file 1 [file pharmaceuticals-16-01465-s001.zip › pharmaceuticals-2573785-supplementary.pdf]

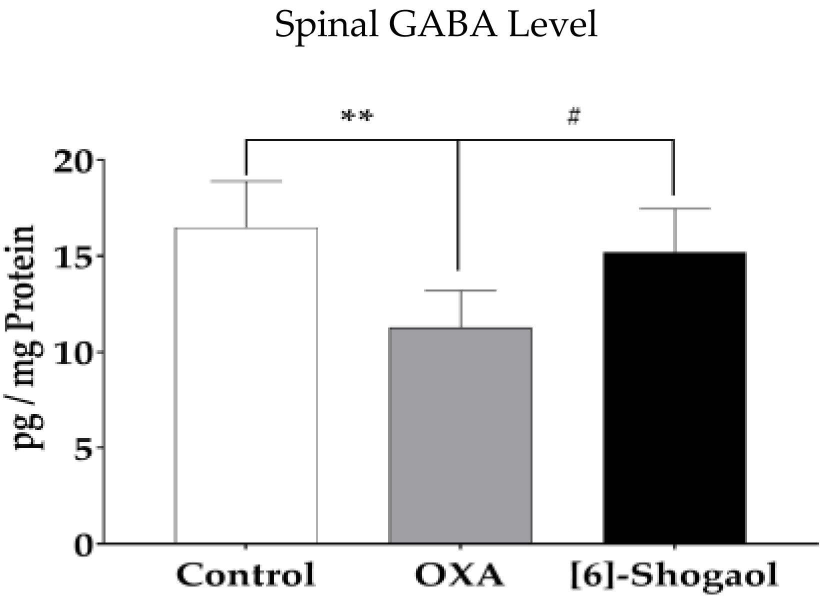

Supplementary Figure S1. The effect of [6]-shogaol on spinal *gamma*-aminobutyric acid (GABA) expression level in oxaliplatin-injected mice. The level of GABA was measured by *enzyme-linked immunosorbent assay (*ELISA). Experiments were conducted on the fourth day prior to the injection of oxaliplatin when allodynia was induced in mice. The lumbar 4-5 spinal cords were sampled for experiment. The control group received 5% glucose. The OXA group received 6 mg/kg of oxaliplatin and 10% of dimethyl sulfoxide (DMSO). The [6]-Shogaol group received oxaliplatin and 10 mg/kg [6]-shogaol. Data are presented as mean ± SD. Control: n = 6; OXA: n = 6; [6]-Shogaol: n = 6. ** $p < 0.01$: Control vs. OXA. # $p < 0.05$: OXA vs. [6]-Shogaol with one-way ANOVA followed by Tukey's multiple comparisons test.
